# Whole-food plant-based Jumpstart for a Deaf and Hard of Hearing cohort

**DOI:** 10.3389/fnut.2023.1125075

**Published:** 2023-04-04

**Authors:** Susan M. Friedman, Kim Scheuer, Beth Garver Beha, Maria Dewhirst, Ted D. Barnett

**Affiliations:** ^1^Department of Medicine, University of Rochester School of Medicine and Dentistry, Rochester, NY, United States; ^2^Rochester Lifestyle Medicine Institute, Rochester, NY, United States; ^3^Plant-Based Telehealth, Austin, TX, United States

**Keywords:** nutrition, prevention, chronic disease, diet, lifestyle, American Sign Language

## Abstract

Deaf and Hard of Hearing (DHH) patients are at high risk of developing chronic illness, and when they do, are at higher risk of poor outcomes than in a hearing community. Rochester Lifestyle Medicine Institute adapted its online, Zoom-based, medically-facilitated 15 Day Whole-Food Plant-Based (WFPB) Jumpstart program, to give DHH participants knowledge, skills, and support to make dietary changes to improve their health. Adaptations included having a medical provider present who is fluent in American Sign Language (ASL), is board-certified in Lifestyle Medicine, and has a Master of Science in Deaf Education; spotlighting participants when asking a question during the Q&A session; using ASL interpreters; utilizing closed captioning/automatic transcription during all Zoom meetings; and employing a Success Specialist to provide outreach *via* text and email throughout the program. Participants had significant positive changes in their eating pattern. They reported improvements in biometric measures as well as in how they were feeling. They all reported that they planned to continue to eat a more WFPB diet than they did prior to Jumpstart. All either agreed or strongly agreed that they learned important information, were confident that they knew the best eating pattern for health, and gained the skills they needed to make changes. Although this was a small pilot program, it suggests that this model can be used to provide education and support for behavior change that will lead to improved health in a DHH community.

## Introduction

Deaf and Hard of Hearing (DHH) patients are at high risk of developing chronic medical conditions through several causal pathways. First, hearing loss can lead to more difficulty with communication and reduced social engagement, resulting in higher risk of loneliness, depression, and dementia (1).

Second, Deaf individuals are almost 7 times more likely than hearing individuals to have inadequate health literacy, which can lead to missed opportunities for health promotion and disease prevention. Factors that contribute to lower health literacy include lack of education, as well as communication issues. The mean reading level has been reported to be grade 5.9; thus, DHH patients often have lower health terminology recognition (2). Variability in communication styles contributes to mixed comprehension: some DHH adults can read English and understand subtitles, others rely on Signed English interpretation by a qualified/licensed interpreter, and yet others rely on American Sign Language, which has its own unique grammar and syntax. Interpreters are costly and finding licensed interpreters is difficult – especially those with skills in the vocabulary and concepts of the medical field.

All of these issues lead to a high prevalence of chronic illness. DHH individuals are at increased risk of having hypertension, hyperlipidemia, pre-diabetes and diabetes, cardiovascular disease, and depression (1, 3).

Additionally, DHH patients experience many barriers to care, and are therefore at high risk for poor medical outcomes once chronic disease is established. Adults who have been deaf since birth or early childhood are less likely to have seen a physician than the general population (4). Cost can be an issue since Deaf individuals are often underemployed and, when employed, earn comparatively less than hearing people, so may struggle to afford traditional and supplementary healthcare programs (5). Suboptimal communication with their providers means that DHH patients are often unaware of their chronic conditions (3) and have poorer physical and mental health compared to hearing populations (6). This, in turn, leads to higher risk of mortality.

According to the American College of Lifestyle Medicine, Lifestyle Medicine (LM) is “a medical specialty that uses therapeutic lifestyle interventions as a primary modality to treat chronic conditions.” LM clinicians “apply evidence-based, whole-person, prescriptive lifestyle change to treat and… often reverse such conditions” (7). LM focuses on six pillars: eating a whole-food, plant-predominant diet, regular physical activity, restorative sleep, stress management, avoiding risky substances, and fostering positive social connections (7). An estimated three-fourths of chronic illness in the US could be avoided if we used these pillars to help patients take charge of their health (8).

Poor diet has been identified as the leading risk factor for mortality and for disability-adjusted life-years since 2010, superseding tobacco use, which had been the leading risk factor prior to that time (9). A whole-food, plant-based (WFPB) diet has been shown to be effective in reducing the impact of many of our most common chronic conditions and diseases, such as hypertension, hyperlipidemia, diabetes mellitus, heart disease, and overweight and obesity (10–13).

Rochester Lifestyle Medicine Institute (RLMI) has developed a 15-day medically-facilitated Jumpstart program that gives participants the knowledge, skills and support to establish a WFPB lifestyle. Previously published results demonstrate that participants experience benefits in a short period of time. Program participants increase their consumption of WFPB components (fruit, vegetables, whole grains, and legumes), and decrease animal products and processed food. As a result, they experience weight loss, and improvements in blood pressure, cholesterol and blood sugar. They also report better energy, sleep, and mood, and less pain (14, 15).

Recognizing the baseline increased risk of chronic disease among DHH individuals, the barriers that they face in healthcare, and the predisposition to poor outcomes, RLMI launched a pilot project to see whether the Jumpstart program could be adapted to a DHH population, whether participants could be recruited, and whether they experienced positive outcomes from the program. The cost of participation in the program was covered through grant funding. This was the first time that this program was adapted for a DHH population, and the first WFPB educational program that we are aware of that has been provided exclusively for a DHH population.

## Context

The Jumpstart program is an online, Zoom-based, medically-facilitated group program. Enrollees in the program take part in 11 h of live, interactive programming over 7 sessions. They receive menus and shopping lists, and participate in a Google Classroom that has additional teaching and support materials. Participants are counseled to eat a low-fat, whole-food plant-based diet, consisting of minimally processed vegetables, fruits, whole grains and legumes. Participants who ask about a minimum number of servings of each component are referred to the advice of Dr. Michael Greger, a nationally recognized expert in WFPB nutrition. Dr. Greger recomends at least 3 daily servings of legumes, 4 servings of whole grains, 5 servings of vegetables, and 4 servings of fruits (16).

Participants are asked to obtain and report baseline fasting cholesterol and glucose tests, as well as biometrics, including weight, blood pressure, and waist circumference. They have frequent opportunities to ask questions of a medical provider throughout the program. They participate in a cooking demonstration on the second day of the program and a “virtual potluck,” in which they send in and then discuss recipes, on the eighth day of the program. Daily emails provide additional support throughout the program. Participants are asked to get follow-up labs and biometrics at the end of the program, and voluntarily share these with RLMI as part of the quality improvement (QI) program.

A convenience sample of 24 adults was recruited from nine states (USA) and Canada. Potential participants were identified through multiple contacts, including DHH networks, a Deaf nutritionist, online communities, word of mouth, non-profit organizations, social media posts, and emails and calls to colleges with large DHH communities. An email template with a coupon code was created, along with a script for phone calls. Calls were made, and targeted emails were sent to administrators of 4 US colleges and 1 high school known for their services for the hearing impaired: Gallaudet University, Washington, DC; Southwest College for the Deaf, Big Spring, Texas; California State University at Northridge, Deaf Studies Department; the National institute for the Deaf at Rochester Institute of Technology, Rochester, NY; and Lexington School for the Deaf, New York, NY. Non-profit organizations with programs for hearing impaired were targeted as well. An event flyer with links to RLMI’s website page, “About Jumpstart,” and an events calendar featuring the DHH cohort were included in correspondence. The Deaf nutritionist and the medical provider for the program both promoted the program widely on their social media channels, mainly Facebook; a preponderance of participants enrolled as a result of this notification. RLMI staff received inquiries from interested parties by email, text, and telephone. They responded to all inquiries with emailed instructions for registration and, when necessary, manually registered participants who needed help.

## Detail

Several adaptations were made to the Jumpstart program in order to facilitate participation from a DHH population (Table 1). A grant enabled the program to be provided free of charge. An information package that included a thorough introduction to WFPB eating, including menus, shopping lists, and helpful hints on food preparation and eating out, was provided 5 days before the start of the program. This gave participants plenty of time to review the information and ask questions early in the program.

**Table 1:** Adaptations to Jumpstart program for a DHH cohort.

Adaptation	Rationale
Program was provided free of charge through grant funding	Many DHH individuals have limited financial resources which limits access
Support materials (Jumpstart guide) sent 5 days before start of program	Provide time to read and absorb teaching materials
Use of American Sign Language interpreters and closed captioning / automatic transcription during all Zoom meetings	Multiple ways to improve communication, so that participants could use preferred method
RLMI Success Specialist outreach by text and email to participants throughout	Provide in-person support; improve feeling of connection to program
DHH participant asking question for Q + A session was spotlighted on Zoom	Improved visibility
Lifestyle Medicine board-certified physician with a Master of Science in Deaf Education provided personalized counseling	Knowledge of barriers; improved communication; improved connection between provider and participant

The medical provider for the program (KS) is board-certified in Lifestyle Medicine, has a Master of Science in Deaf Education, is fluent in American Sign Language (ASL), and understands the culture of the Deaf community. She is hearing, with Deaf and Hard of Hearing family members. Before becoming a practicing physician, she taught Deaf and Hard of Hearing students in multiple different schools in the United States and other countries. She has been a professional interpreter in many settings in the United States and internationally.

Participants in the program were asked to raise their electronic hands so that they could be spotlighted when asking a question during the Q&A session, providing better visibility for the other participants. Multiple ways of communicating were employed, including use of ASL and closed captioning/automatic transcription during all Zoom meetings. For any session lasting more than 1 h, 2 interpreters participated, to allow them time to rest, as it is physically taxing to interpret for more than an hour at a time. On days of small group “breakout” sessions, several interpreters participated so that one interpreter was available for each breakout room. All ASL interpreters were Board Certified Interpreters who had passed the National Interpreter Certification (NIC) test that is given jointly by the National Association of the Deaf (NAD) and the Registry of Interpreters for the Deaf (RID). Finally, a Success Specialist provided outreach *via* text and email throughout the program, for additional support.

Permission was given from the University of Rochester Institutional Review Board to use the data collected from RLMI as part of the QI program. The study was considered exempt.

Forty-two people registered for the program, and 24 attended. Of those, 18 provided one or more responses to questions. Changes reported from the beginning to the end of the Jumpstart were compared *via* 2-tailed paired t-tests.

The 11 participants who provided pre-post data to the QI program reported significant changes in their dietary pattern (Table 2). They ate more vegetables, leafy greens, fruit, and legumes, and less meat, eggs, added fat, processed food, and high-fat plant food. In rating how adherent their eating patterns were to a very low-fat WFPB diet, participants reported an increase from baseline to the end of the program of 4.55 points, from 4.36 to 8.91 on a scale from 1 (not very low fat WFPB at all) to 10 (every meal very low fat WFPB).

**Table 2:** Changes in dietary components* during the Jumpstart program (*n* = 11).

	Beginning of program (mean (SD))	End of program (mean (SD))	*p* value
*Measure (servings)*			
Vegetables	4.66 (5.78)	7.27 (6.14)	0.003
Leafy greens	1.96 (2.73)	3.98 (2.41)	<0.001
Fruit	2.80 (2.99)	4.64 (2.95)	<0.001
Whole grains	3.05 (3.42)	3.38 (2.43)	0.65
Legumes	1.10 (1.49)	2.35 (1.43)	<0.001
High-fat plant foods	3.13 (2.77)	0.82 (2.09)	<0.001
Meat	1.09 (0.94)	0 (0)	0.003
Eggs	1.64 (1.86)	0.27 (0.47)	0.01
Added fat	2.00 (1.77)	0.09 (0.30)	0.003
Processed food	2.18 (1.33)	0 (0)	<0.001
Rating of adherence^**^	4.36 (3.01)	8.91 (1.38)	0.001

* Participants are counseled to eat a very low-fat, whole-food plant-based diet, consuming more vegetables, leafy greens, fruit, whole grains, and legumes, and no high-fat plant foods, meat, eggs, added fat and processed food.

** Rated adherence to a very low-fat whole-food plant-based diet on a scale from 1-10.

Participants who provided pre-post data also had changes in clinical and self-reported measures (Table 3). They lost weight, and had a decrease in pulse rate and systolic blood pressure, as well as in total and LDL cholesterol. They reported improvements in energy, sleep, and mood. Of the 11 participants who provided a response, all of them reported that they planned to continue to eat a more WFPB diet than they did prior to Jumpstart. All either agreed or strongly agreed that they learned important information, were confident that they knew the best eating pattern for health, and gained the skills they needed to make changes (Table 4).

**Table 3:** Jumpstart outcomes.

	*n*	Beginning of program (mean (SD))	End of program (mean (SD))	*p* value
*Biometric measures*				
Weight (pounds)	10	172.0 (43.5)	166.8 (41.9)	0.004
Waist circumference (inches)	8	39.1 (6.3)	37.1 (4.5)	0.13
Heart rate (beats per minute)	10	76.3 (14.8)	69.3 (9.9)	0.02
Systolic blood pressure (mm Hg)	7	129.9 (13.5)	117.4 (11.3)	0.01
Diastolic blood pressure (mm Hg)	7	76.7 (7.7)	76.6 (6.7)	0.95
Cholesterol (mg/dL)	6	204.2 (39.0)	167.2 (21.3)	0.03
LDL cholesterol (mg/dL)	6	122.8 (25.5)	100.8 (22.1)	0.03
HDL cholesterol (mg/dL)	6	67.5 (43.2)	54.7 (31.4)	0.10
Triglycerides (mg/dL)	6	139.5 (72.1)	110.5 (20.5)	0.36
Glucose (mg/dL)	4	86.8 (3.3)	88.5 (2.1)	0.24
*Self-reported measures* ^*^				
Energy	11	5.55 (2.70)	8.27 (1.56)	0.004
Sleep	11	6 (3.03)	7.91 (1.81)	0.048
Mood	11	5.91 (2.55)	8.18 (1.40)	0.004
Pain	11	3.36 (2.50)	3.27 (2.53)	0.90

* Rated on a scale from 1–10. Energy, sleep, and mood are rated from 1 (very poor) to 10 (excellent). Pain ranges from 1 (no pain) to 10 (constant severe pain).

**Table 4:** Participants’ perceptions of program benefit (*n* = 11).

	Strongly agree (%)	Agree (%)
“I am confident that I know about the type of eating pattern that is best for my health.”	54.5	45.5
“I learned important information about the role of nutrition in health.”	90.9	9.1
“I gained the skills I need to make changes to my health.”	81.8	18.2

## Discussion

This pilot program demonstrates that an online medically-facilitated program designed to give participants the knowledge, skills and support to develop a WFPB lifestyle, can be adapted for a DHH cohort. Despite multiple potential barriers in this population, participants were identified and recruited from several networks across North America. Participants made changes to their eating pattern and expressed an intent to continue to eat a more healthy diet following the program. They noted positive changes – both in how they were feeling, and in biometric measures. Finally, they reported that they got information and gained skills needed to improve their health.

Although we are not aware of other programs that have provided education to a DHH community to help them adopt a WFPB lifestyle, many interventions have been developed to try to reduce barriers and improve access to health education in the Deaf community. A recent systematic review demonstrated that several innovations led to improved outcomes, and could serve to reduce health inequities. These included the use of online interventions, and using Sign Language during telehealth visits. However, it was noted that stronger evidence was needed to assess these interventions (6).

The adaptations to the program included multiple accommodations to improve communication, connection, and comprehension. Communication was enhanced in multiple ways: by using ASL interpreters and closed captioning/automatic transcription during all Zoom meetings, spotlighting participants during Q&A for better visibility, and having a Lifestyle Medicine physician who had a Master of Science in Deaf Education for personalized counseling. Connection was enhanced through a Success Specialist who communicated *via* email and text. Finally, comprehension was improved through giving 5 days of lead time to review written materials; multimodal communication as described above; and email responses to individual questions.

An in-person variation of this program could help to promote engagement and social support. Prior to the pandemic, the Jumpstart was run exclusively as an in-person program. Since the pandemic, all programs are run online, which enables reaching a larger and more geographically diverse group. An adaptation of the program is being developed using a shared medical visit approach, so it is feasible that practitioners with a large DHH cohort of patients could run the program in person.

Feedback was obtained through standard questionnaires on Day 15 of the program. These questionnaires are sent to all participants in the Jumpstart program, and questions were not specific to this cohort. When asked what the program could do to help participants stay on the path of a WFPB diet, responses from this cohort included sending out recipes, occasional check-in reminders, and availability of ongoing support, including a maintenance program and opportunities to communicate with program staff. When asked about barriers, none of the respondents mentioned communication issues. More qualitative feedback from DHH participants would be helpful to improve future iterations of a DHH WFPB program.

## Limitations

Despite the adjustments to make the program accessible, the team still encountered some issues in running the program. Some participants preferred the interpreters to use American Sign Language, and felt the interpreters were not as skilled as desired in that language. Those participants noted that the interpreters were using a pidgin of signed English instead of ASL, which has a different and distinct grammar and syntax, making it much harder to understand (similar to someone interpreting from the French language to English by putting the English words into a French word order making the interpretation challenging to understand).

Some individuals signed up but did not participate. Since the cost of attending was covered by a grant, potential participants did not have a financial incentive to follow through with the program. Future iterations of this program could consider charging a nominal amount, so that participants are not limited by financial barriers, but still have “skin in the game” to follow through.

This was a small pilot study, designed as a “proof of concept,” to demonstrate that an online Zoom-based medically-facilitated Lifestyle Medicine program with previously proven benefits could be adapted to an underserved community with barriers in both access and communication. Because this was not designed as a prospective clinical trial, demonstration of impact is limited to the responses that were voluntarily provided through an ongoing QI program. It is certainly possible that the participants who had less favorable results did not respond to the surveys.

Additionally, we used a “post-pre” analysis, in which participants were able to compare their baseline responses to their follow up. This was done so that participants could assess change during the 15 days (e.g., changes in pain or energy), and because the way in which participants measure certain outcomes (e.g., how whole-food plant-based their diet is) may change over the course of the 15 days. This approach, as well as a pre-post approach, are vulnerable to a “social desirability” bias of providing responses that are acceptable; however, this is generally a limitation of self-reported measures (17).

A larger, prospective study would reduce these biases, and yield more generalizable results. In addition, it could provide opportunities to evaluate the characteristics that are associated with positive results. However, the breadth of positive results reported by this cohort suggests that this is a model that can be used to provide education and support for behavior change that will lead to improved health in a DHH community.

## Conclusion

This pilot study provides a framework for adapting an online medically-facilitated program to educate a traditionally underserved community with multiple barriers to care –namely, a Deaf and Hard of Hearing cohort –giving them knowledge, skills and support to adopt a WFPB lifestyle. Preliminary findings suggest that participants are able to make significant changes, leading to short-term health improvements. Further large-scale, prospective studies are needed to rigorously evaluate this approach, building on the “lessons learned” from the pilot program.

## Data availability statement

The data analyzed in this study is subject to the following licenses/restrictions: The de-identified dataset is a subset of a quality improvement dataset managed by Rochester Lifestyle Medicine Institute. Requests to access these datasets should be directed to Bruce.pollock@roclifemed.org.

## Ethics statement

The study involving human participants was reviewed and approved by the University of Rochester School of Medicine and Dentistry Institutional Review Board. Written informed consent for participation was not required for this study in accordance with national legislation and institutional requirements.

## Author contributions

SF: analysis, drafting, and revision of manuscript. KS and TB: program conceptual framework, review, and revision of manuscript. BB and MD: program conceptual framework, recruitment of participants, review, and revision of manuscript. All authors contributed to the article and approved the submitted version.

## Conflict of interest

The authors declare that the research was conducted in the absence of any commercial or financial relationships that could be construed as a potential conflict of interest.

## Publisher’s note

All claims expressed in this article are solely those of the authors and do not necessarily represent those of their affiliated organizations, or those of the publisher, the editors and the reviewers. Any product that may be evaluated in this article, or claim that may be made by its manufacturer, is not guaranteed or endorsed by the publisher.
